# Bee venom acupuncture for adhesive capsulitis: A protocol for systematic review and meta-analysis

**DOI:** 10.1097/MD.0000000000019975

**Published:** 2020-05-01

**Authors:** Xiaohua Chen, Huaying Fan, Jiao Chen, Huayu Fan, Ping Wu

**Affiliations:** a Department of Central Transportation Center, West China Hospital, Sichuan University; b College of Acupuncture and Tuina, Chengdu University of Traditional Chinese Medicine; c Respiratory Failure Center and Lung Transplant Unit, Sicuhan Province Hospital, Chengdu City, Sichuan Province, China.

**Keywords:** bee venom acupuncture, protocol, shoulder adhesive capsulitis, systematic review

## Abstract

**Background:**

Bee venom acupuncture has been used in treating patients with shoulder adhesive capsulitis, yet the effectiveness and safety remains unclear. Therefore, this systematic review will aim to assess the effectiveness and safety of bee venom acupuncture for shoulder adhesive capsulitis.

**Methods:**

Electronic databases including EMBASE, PUBMED, the Cochrane Central Register of Controlled Trials, China National Knowledge Infrastructure, Chinese Scientific Journal Database, Wanfang Database, and Chinese Biomedical Literature Database will be searched for relevant randomized controlled trials from their inception to the search data without language and publication status. Randomized controlled trials involving bee venom acupuncture for treating shoulder adhesive capsulitis will be included. The primary outcome will be pain visual analogue scale, and secondary outcomes include active and passive range of motions, shoulder pain and disability index. Meta-analysis will be conducted using Review Manager software (V.5.3). The results will be presented as risk ratio for dichotomous data, and standardized or weighted mean difference for continuous data.

**Results:**

The results will be disseminated through a peer-reviewed journal publication.

**Conclusion:**

These systematic review findings will provide an evidence of bee venom acupuncture for shoulder adhesive capsulitis, and help to inform clinical practitioners and policy-makers in the decision-making.

**Ethics and dissemination:**

Ethics approval and patient consent are not required as this study is a systematic review based on published articles.

## 1 Introduction

Shoulder adhesive capsulitis, also known as frozen shoulder, is a common shoulder condition caused by injury or degeneration of the shoulder joint and articular capsule.^[1]^ The condition is characterized by pain, stiffness, and dysfunction of the affected shoulder.^[2]^ The prevalence of shoulder adhesive capsulitis is about 2% to 5% in general adult population and 10% to 20% in diabetics of the general population.^[3]^ The majority of patients diagnosed with adhesive capsulitis are women between 40 and 60 years of age.^[4]^ Patients with shoulder adhesive capsulitis are more likely to experience sleepless, anxiety, and even disability, which may seriously affect the patients’ daily life and occupational activities.^[5]^ As a self-limited disease, shoulder adhesive capsulitis can persist for years with some patients never regaining full function of their shoulder, which may account for an enormous economic pressure on healthcare.^[6]^ In the United Kingdom, for example, the National Health Service (NHS) costs at least £44.1 million to £110.3 million based on a single general practitioner consultation for each case.^[7]^ Currently, the treatments of shoulder adhesive capsulitis include cortisone injections, nonsteroidal anti-inflammatory drugs (NSAIDs), physiotherapy, arthroscopic capsular release and manipulation under anesthesia, etc.^[6,8,9]^ However, most of the treatments only have some effective therapy in a short-term,^[10–12]^ and the majority of these interventions are often accompanied by varying degrees of side-effects.^[13]^

Bee venom acupuncture is a form of acupuncture in which bee venom is applied to the tips of acupuncture needles, stingers are extracted from bees, or bees are held with an instrument, such as a forceps, squeezed to cause the stinger to emerge from the lower abdomen, and then either the needles or stinger is applied acupoints on the skin.^[14]^ Bee venom acupuncture has been widely used as a complementary and alternative therapy to relieve pain and inflammation for 3000 years.^[15,16]^ Studies have demonstrated that bee venom acupuncture contains many kinds of components which may show pharmacological actions such as anti-inflammation, anti-apoptosis, anti-fibrosis and anti-arthrosclerosis.^[17]^ With these pharmaceutical characteristics, bee venom acupuncture has been used as the therapeutic method in treating osteoarthritis keen pain,^[18]^ Parkinson's disease,^[19]^ rheumatoid arthritis,^[20]^ etc. Moreover, studies have indicated that bee venom acupuncture with physical therapy can be more effective in improving pain and function than physical therapy alone, and may help to improve long-term of life in patients with shoulder adhesive capsulitis.^[21,22]^

Although bee venom acupuncture has been utilized to treat shoulder adhesive capsulitis for a long time, there was no systematic review conducted to assess the effectiveness and safety of bee venom acupuncture for shoulder adhesive capsulitis. Therefore, we conducted this systematic review to evaluate the effectiveness and safety of bee venom acupuncture for shoulder adhesive capsulitis.

## 2 Methods

### 2.1 Protocol register

The protocol of this review has been prepared under the guidance of Preferred Reporting Items for Systematic Review and Meta-Analyses Protocols guidelines, and it has been registered on OSF platform (https://osf.io/registries) with a registration number 10.17605/OSF.IO/E6A5 V.

### 2.2 Ethics

As all eligible studies were approved by local institution review boards and ethical committees, this study requires no ethical approval.

### 2.3 Information sources and search strategy

The following seven electronic databases including EMBASE, PUBMED, the Cochrane Central Register of Controlled Trials (CENTRAL), China National Knowledge Infrastructure (CNKI), Chinese Scientific Journal Database (VIP database), Wanfang Database, and Chinese Biomedical Literature Database will be searched for relevant RCTs from their inception to the search data without language and publication status. The search terms will consist of three parts: bee venom acupuncture, shoulder adhesive capsulitis, and randomized controlled trial (RCT). The detailed search strategy used in PUBMED is presented in Table 1.

**Table 1:**
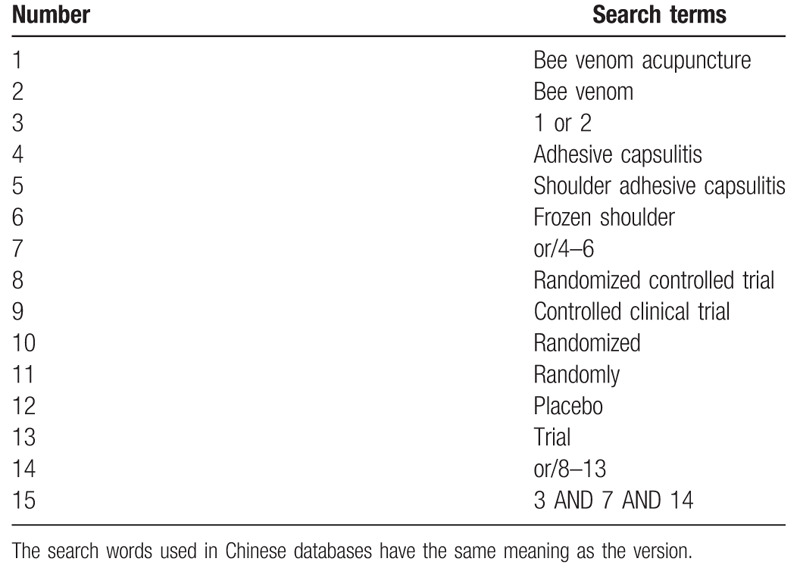
Search strategy used in PUBMED database.

### 2.4 Eligibility criteria

#### 2.4.1 Study design

All RCTs involving bee venom acupuncture for treating shoulder adhesive capsulitis will be included without language or publication restriction. Studies with non-RCT design, quasi-experiment design, unobtainable data, duplicate publications, animal experiments, and reviews or case reports will be excluded.

#### 2.4.2 Participants

Adult participants (≥18 years old) presenting with shoulder adhesive capsulitis will be included regardless of sex, race, or educational and economic status. Participants with severe physical or mental disease will be excluded.

#### 2.4.3 Interventions and comparators

Interventions in the treatment group will include bee venom acupuncture. Comparators will include physiotherapy, placebo control, no treatment/waiting list control, or active treatment (e.g., nonsteroidal anti-inflammatory drugs and/or local injection of corticosteroids, etc.) will be included. RCTs evaluating bee venom acupuncture combined with another treatment compared with that other treatment alone will also be included.

#### 2.4.4 Outcomes

Primary outcome will be the pain visual analogue scale (VAS). Secondary outcomes will include active and passive range of motions (ROMs), shoulder pain and disability index (SPADI). Besides, safety measurement and adverse events will also be considered.

### 2.5 Selection of studies and data extraction process

**Figure 1:**
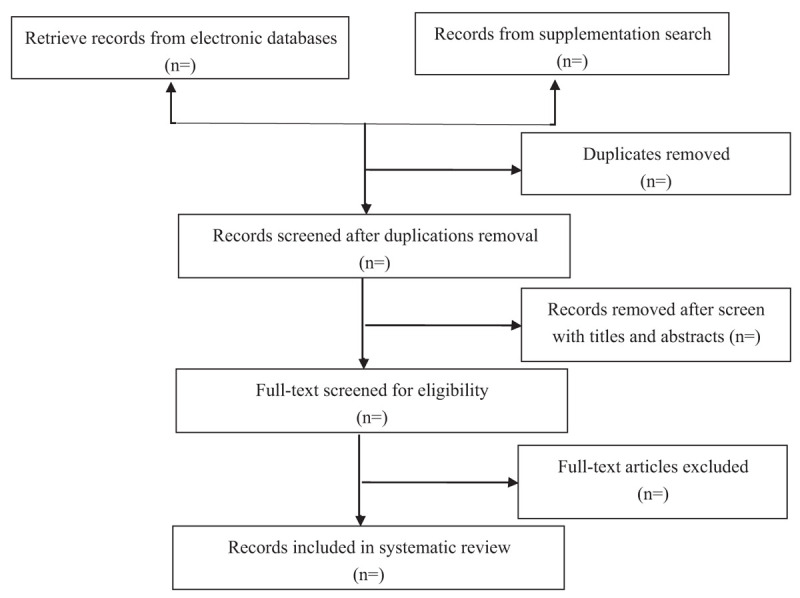
The flow chart of the systematic review. This flow chart is based on PRISMA framework, which shows the whole process of literature search in the study, including records retrieving, screening, inclusion, exclusion with reasons and articles included in final qualitative and quantitative analysis.

Two reviewers (HYF and XHC) will, respectively, screen the study titles and abstracts to identify potentially eligible studies according to the inclusion criteria. The full texts reviews will be obtained and independently screened before final inclusion. Two reviewers (JC and HYF) will independently extract the data of all eligible studies by employing a standard pre-designed form. The following items will be included in the data extraction form: first author, year of publication, country, number of centers and the participants, study design, number of groups, interventions, comparisons, outcomes (primary and second outcomes), and conclusions. The final extracted data will be cross-checked and any disagreement regarding to the extracted data will be discussed and adjudicated by a third reviewer (PW). If any data are insufficient or unclear, the first or corresponding author for the study concerned will be contacted via e-mail or telephone to provide additional information. The flow chart based on PRISMA^[23]^ is displayed in Figure 1.

### 2.6 Risk of bias assessment

Two reviewers (XHC and JC) will independently evaluate the risk of bias of each study included according to the Cochrane Collaboration Risk of Bias assessment tool, which focuses on six domains: random sequence generation, allocation concealment, blinding, incomplete outcome data, selective reporting, and other bias. The assessments will be categorized into three levels of bias: low risk, high risk, or unclear risk. Any disagreement between 2 reviewers will be resolved by discussion or the introduction of a third reviewer (PW).

### 2.7 Data synthesis

Meta-analysis will be done by using Review Manager software (V.5.3). Mean difference (MD) with 95% confidence intervals will be used for continuous data and risk ratios (RRs) will be used for dichotomous data. Random effect model will be selected in data pooling. Heterogeneity will be assessed by chi-square and *I*^2^ test. If heterogeneity is found (*I*^2^ > 50%), we will try to explain the clinical and methodological diversity or conduct subgroup analysis. However, if we cannot determine the reason for heterogeneity, we will conduct a descriptive analysis instead of a meta-analysis.

### 2.8 GRADE quality assessment

Grading of Recommendations Assessment, Development and Evaluation (GRADE), which is a method of grading the level of evidence and is developed by the GRADE Working Group, will be applied to evaluate the quality of evidence by two independent reviewers (HYF and HYF). The GRADEpro software (version 3.6 for Windows, Grade Working Group) will be utilized.

## 3 Discussion

Shoulder adhesive capsulitis is a common disorder that causes pain and function impairment of shoulder. Bee venom acupuncture has been used to treat a number of musculoskeletal diseases such as lumbar disc disease, osteoarthritis of knee, rheumatoid arthritis, adhesive capsulitis and lateral epicondylitis. Studies have shown that bee venom acupuncture may be an effective way in treating shoulder adhesive capsulitis. Given that systematic reviews with good quality can help provide best evidence in clinical practice, we design this study protocol. Hopefully, it can do benefit to the clinical decisions and can improve the treatment effect.

## Author contributions

**Conceptualization:** Xiaohua Chen, Huaying Fan

**Data curation:** Jiao Chen, Ping Wu

**Formal analysis:** Jiao Chen, Huaying Fan

**Investigation:** Huayu Fan

**Methodology:** Xiaohua Chen, Jiao Chen

**Resources:** Huayu Fan, Xiaohua Chen

**Software:** Jiao Chen, Huaying Fan

**Supervision:** Ping Wu

**Writing – original draft:** Xiaohua Chen, Huaying Fan

**Writing – review & editing:** Ping Wu, Jiao Chen
